# Novel Criteria for Intratumoral Budding with Prognostic Relevance for Colon Cancer and Its Histological Subtypes

**DOI:** 10.3390/ijms222313108

**Published:** 2021-12-03

**Authors:** Pantea Pour Farid, Markus Eckstein, Susanne Merkel, Robert Grützmann, Arndt Hartmann, Volker Bruns, Michaela Benz, Regine Schneider-Stock, Carol I. Geppert

**Affiliations:** 1 Experimental Tumorpathology, University Hospital, Friedrich-Alexander-University of Erlangen-Nüremberg (FAU), 91054 Erlangen, Germany; panteap96@gmail.com (P.P.F.); regine.schneider-stock@uk-erlangen.de (R.S.-S.); 2 Institute of Pathology, University Hospital, Friedrich-Alexander-University of Erlangen-Nüremberg (FAU), 91054 Erlangen, Germany; markus.eckstein@uk-erlangen.de (M.E.); arndt.hartmann@uk-erlangen.de (A.H.); 3 Comprehensive Cancer Center-EMN (CCC), University Hospital, Friedrich-Alexander-University of Erlangen-Nüremberg (FAU), 91054 Erlangen, Germany; susanne.merkel@uk-erlangen.de (S.M.); robert.gruetzmann@uk-erlangen.de (R.G.); 4 Department of Surgery, University Hospital, Friedrich-Alexander-University of Erlangen-Nüremberg (FAU), 91054 Erlangen, Germany; 5 Fraunhofer Institute for Integrated Circuits IIS, Am Wolfsmantel 33, 91058 Erlangen, Germany; volker.bruns@iis.fraunhofer.de (V.B.); michaela.benz@iis.fraunhofer.de (M.B.)

**Keywords:** tumor budding, prognostic biomarker, metastasis, immunohistochemistry, survival analysis, next-generation tissue microarray analysis, medullary carcinoma, mucinous carcinoma, signet ring cell carcinoma, histological subtypes

## Abstract

Peritumoral budding and intratumoral budding (ITB) are important prognostic factors for colorectal cancer patients. Scientists worldwide have investigated the role of budding in tumor progression and its prognosis, but guidelines for reliably identifying tumor buds based on morphology are lacking. In this study, next-generation tissue microarray (ngTMA^®^) construction was used for tumor bud evaluation, and highly detailed rule-out annotation was used for tumor definition in pancytokeratin-stained tissue sections. Initially, tissues of 245 colon cancer patients were evaluated with high interobserver reliability, and a concordance of 96% was achieved. It was shown that high ITB scores were associated with poor distant metastasis-free survival (*p* = 0.006 with a cut-off of ≥10 buds). This cut-off was defined as the best maximum value from one of two/three ngTMA^®^ cores (0.6 mm diameter). ITB in 30 cases of mucinous, medullary, and signet ring cell carcinoma was analyzed for the subsequent determination of differences in tumor bud analyses between those subtypes. In conclusion, blinded randomized punched cores in the tumor center can be useful for ITB detection. It can be assumed that this method is suitable for its adoption in clinical routines.

## 1. Introduction

Colorectal cancer (CRC) is one of the most aggressive cancers worldwide in terms of its incidence and mortality. Despite the widespread implementation of molecular and diagnostic screening programs and modern imaging techniques, patient outcomes are still poor, especially in the case of metastases [1,2,3]. Tumor budding seems to be a relevant independent factor for the prognosis of patients with colorectal cancer (CRC). It is also based on morphology and seems to outperform evaluations based on the WHO grade [4,5]. The availability of novel diagnostic markers that allow for the prediction of prognosis and the risk of metastasis could help clinicians in tailoring therapeutic strategies. Therefore, we believe that novel and more detailed criteria for tumor budding should be integrated into routine diagnostics.

In 1949, tumor budding was described as “sprouting” for the first time [6]. Histologically, tumor buds were defined as both single cells and a group of fewer than four or five cells [7,8,9,10,11] and have been assessed by the community through both classical HE staining and immunohistochemistry (IHC) [4,9,11,12]. Tumor budding appears to be correlated with lymphatic and venous invasion as well as lymph node metastases [12]. This was also illustrated by Hiroshi Kawachi et al. in 2015 in a multicenter setting [13]. A higher number of tumor buds is correlated with a worse prognosis not only in CRC but also in laryngeal carcinoma, breast cancer, oral squamous cell carcinoma, and other types of cancer [14,15,16].

Tumor budding was predominantly described for the tumor invasion front, being a clear sign of metastatic dissemination [4,11,13]. Thus, these disseminating small tumor cell aggregates were associated with the epithelial-to-mesenchymal transition (EMT), a fundamental process in embryogenesis and metastasis [17,18]. The gene signature of tumor buds resembles the intermediate EMT type and was referred to as “EMT-like” [18,19]. The invasion pattern and tumor morphology and, thus, tumor budding, especially at the invasion front, are strongly influenced by the immune system [5,20,21].

In 2015, an expert panel of pathologists in the field of tumor budding organized the first International Tumor Budding Consensus Conference (ITBCC), with the goal of generating evidence-based guidelines for tumor budding. Details of many different aspects, such as the definition of tumor buds, recommended staining procedures, and clinical relevance were discussed and summarized in a consensus statement [4]. However, there is still no general acceptance towards including bud scoring as a diagnostic and clinically relevant factor in daily routines, as described by Mitrovic et al., who pointed out various reasons for this [20]. For more details regarding E-cadherin as a biomarker, EMT, and the immune response, see Sections S1–S3.

In this study, we aimed to create a unified definition of ITB that might also be applicable to different histological subtypes based on criteria that have not been established in previous studies [11,21]. Our criteria were established based on immunohistochemically stained tissue slices that provided better conditions for tumor budding evaluation [22]. We identified a high prognostic value of ITB based on ngTMAs^®^ in our CRC cohort. We have created an easy-to-follow protocol for the annotation of tumor buds that is practicable in clinical routines.

## 2. Results

### 2.1. The Cohort of Patients with Adenocarcinoma

Peritumoral and intratumoral budding in colorectal cancer was retrospectively analyzed in ngTMA^®^ punches of 245 NOS (not otherwise specified) adenocarcinoma patients. The term NOS describes a classically intestinal and glandular differentiated adenocarcinoma. All clinicopathological factors are given in detail in Table 1. Patients who have already metastasized at diagnosis are classified as pM1. However, these were resected to R0 and were thus pooled with the other pM0 patients. If these patients developed a secondary metastasis over time, they were included in the distant metastasis-free survival group in the same way as the no primary metastatic patient group who developed a metastasis over time.

### 2.2. ITB Evaluation and the Criteria Definition

**Figure 1:**
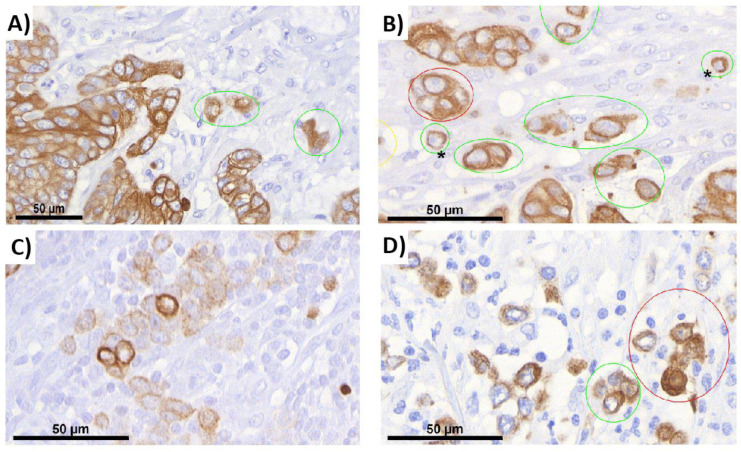
Budding analysis—cell properties. Histological specimens were stained using antibodies AE1/AE3 according to the manufacturer’s recommendations. They were scanned using 3DHISTECH PANNORAMIC 250 Flash II with a CIS VCC camera (3DHISTECH Ltd., Budapest, Hungary) and a 40× Plan-Apochromat objective (Karl Zeiss, Jena, Germany) and a resolution of 0.194 µm/pixel, scan compression setting 80 jpg. The digital slides were analyzed using CaseViewer (Ver. 2.4., 3DHISTECH Ltd., Budapest, Hungary). Tumor buds were marked in pictures using the annotation tool in CaseViewer. The different histological aspects of cell properties that were important for analyzing buds can be seen in (**A**–**D**). The cells marked with a green circle were those considered as real buds, whereas cells marked with a red circle did not fulfill the criteria to be considered buds. (**A**) Low intensity staining: the cells on the left side marked in green have a lower staining intensity than the cells on the right. There are weakly stained cells in the tumor. Therefore, the staining intensity of cells on the left side is an average of the staining intensity of the whole tumor, and they were considered as buds on this basis. Magnification: 62.1×. (**B**) Incomplete circumference: the cells marked with (*) do not have a continuous circumference. They were still considered buds because the circumference exceeds 50% of the cell. Morphologically, the cell is very similar to a tumor cell. It has a light halo and has a size comparable to the average cell size of the rest of the surrounding tumor cells. Magnification: 102.8×. (**C**) Positive and negative cells: as shown, strongly stained cells can be found at the center of a group of many negative cells. Positive cells can mistakenly be evaluated as real buds. The negative cells must also be considered and are very close. They form a unit together with the strongly stained cells and thus exceed the maximum count of 4 for a real bud. Therefore, the strong positive cells are not considered a bud. Magnification: 102.8×. (**D**) Cell count limit: the image shows the limit for maximum cell numbers at which a cell group is no longer considered a bud (>4 cells). The cell group marked in green consists of four cells and thus fulfills the criterion regarding the maximum cell count of a bud (considering distance to other groups of cells). The cell group marked in red consists of five cells. This exceeds the maximum number and, thus, cannot be considered a bud. Magnification: 102.8×.

**Figure 2:**
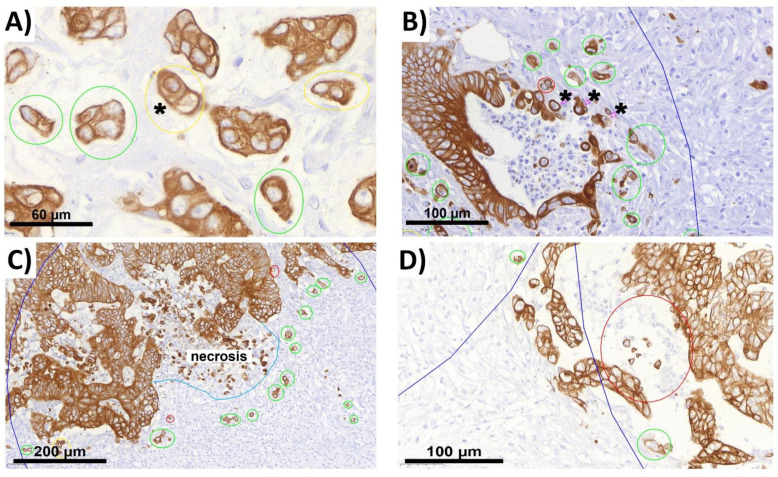
Budding analysis depending on cell environment (**A**–**D**). For technical data, see legend in Figure 1. (**A**) Distance between cells: the distances between the questionable bud (*) and the tumor cell group next to it is shorter than the average diameter of the cells of these two. The questionable bud is therefore considered as a unit with the tumor cell group next to it. A group of more than 4 cells is formed and thus is not considered a bud. Magnification: 78.8×. (**B**) Ruptured gland with comedonecrosis: the marked cells (*) are too close to the comedonecrosis, which is why they cannot be considered buds that are part of the necrosis. The cells marked in green are not associated with comedonecrosis and are considered buds. Magnification: 37.4×. (**C**) Necrosis border: the border of necrosis is marked in blue. No tumor cells in this area can be considered buds. All cells marked in green are sufficiently far away from this area, so they are considered buds. Magnification: 18.0×. (**D**) Intraluminal location: the cells marked in red are located in the lumen of the gland and, therefore, cannot be considered buds. Buds have to be located extraluminally in the connective tissue. Magnification: 38.9×.

In the process of our evaluation, we were able to expand on the previous criteria for tumor budding reported in the literature with more precise definitions. We identified different criteria for ITB in terms of the cell properties (Figure 1) and environment (Figure 2) and compiled them in a flowchart (see Section 4.4). By analyzing 14,530 buds, we were able to adapt and further improve these annotations over time. During this process, the importance of morphology in bud evaluation became increasingly clear to us. Tumor budding, like other morphology-based aspects in general, is dependent on the experience of the observer. It became obvious that there is no single criterion that can clearly be used to directly define a bud on its own.

It must be considered that pancytokeratin staining highlights intracellular intermediate filaments. To date, no suitable immunohistochemical staining has been proven to unambiguously label tumor buds in literature. AE1/AE3 and various cytokeratin staining methods have been used so far. Accordingly, pancytokeratin does not stain the cell membrane. A stained circumference of the cell is therefore an artifact and results from the asymmetric distribution of cytoplasm around the nucleus.

Furthermore, loss of pancytokeratin or a weak staining may be due to EMT. This may be another reason why weakly stained bud candidates should be considered for ITB.

### 2.3. The Complicating Aspects in Budding Evaluations: Cell Truncation

**Figure 3:**
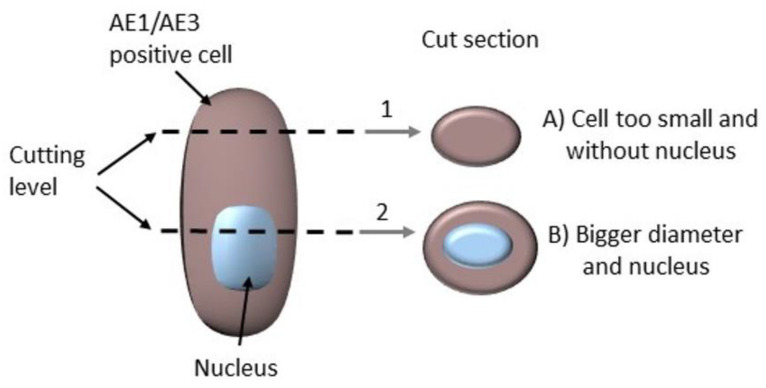
Scheme for cell truncation (for AE1/AE3). Cell components are stained differently: in Cutting Level 1 with positive cytoplasm and without a representative nucleus (negative) (**A**) and in Cutting Level 2 with a representative nucleus (**B**). Nevertheless, this effect occurs in all section preparations, including HE, and thus always influences the interpretation of morphology in any staining.

Section artifacts are a general problem in evaluating morphology in tissue sections. Therefore, cell truncation must be considered because, depending on the section level, cells may be cut peripherally and may appear not to have representing nuclei and, therefore, appear smaller than surrounding cells (Figure 3). This can lead to true buds being rejected because they seemingly do not fulfill the necessary criteria. Similar results have been obtained in the FISH analysis [23]. Ultimately, IHC analyses are FFPE-based, and morphology always depends on sectioning thickness [24]. In the course of the evaluation, some obvious bud candidates that fulfilled all relevant criteria showed no explicit nucleus but were evaluated regarding their tumor morphology by taking cell/nuclear truncation effects into account. This affected only a small subset of bud candidates.

### 2.4. Peritumoral Budding (PTB) in NOS Adenocarcinoma

We also evaluated our TMAs at the invasion front using the same principle and did not obtain a clear cut-off or any significant correlation with clinicopathological parameters. In contrast to the above-mentioned studies, we evaluated blind punched areas on the invasion front rather than targeted hot spots. Due to the decreased ambiguity of ITB, we further focused on the tumor center. By contrast, this region seems better for evaluation because the stronger effects of the immune system, such as by cytotoxic T-cells, have been observed at the invasion front [25].

### 2.5. Intratumoral Budding in NOS Adenocarcinoma and Clinical Outcomes

**Figure 4:**
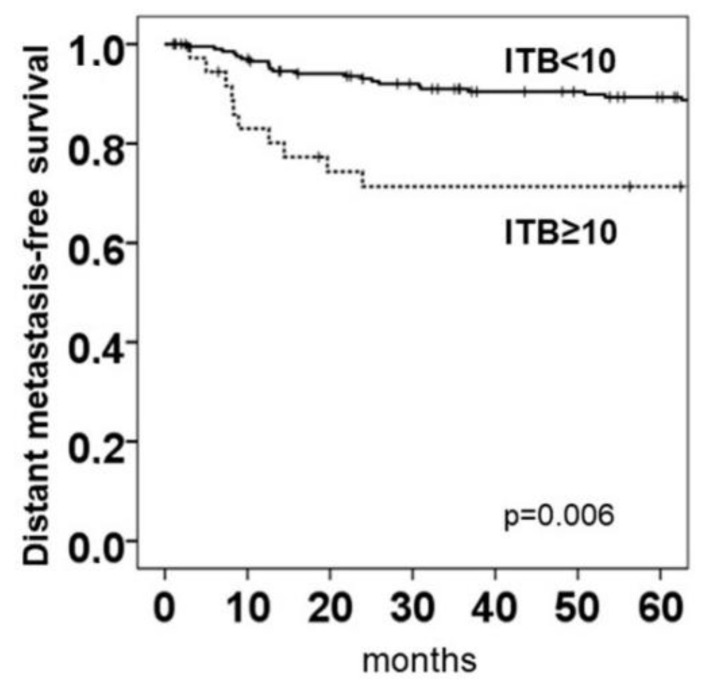
Kaplan–Meier analysis for NOS adenocarcinoma (*n* = 245). Both primary pM1 patients (with local and distant R0 resection, *n* = 6) and primary pM0 patients developed secondary metastases (*n* = 29). Prognostic significance of ITB score and distant metastasis-free survival. Log-rank test calculated with SPSS Version 26, IBM (Armonk, New York, NY, USA).

The Kaplan–Meier plots for counting off intratumoral buds in ngTMA^®^ using pancytokeratin staining revealed a cut-off of ≥10 buds to be unfavorable for distant metastasis-free survival (*p* = 0.006, cut-off ≥10 buds; Figure 4). The cut-off was defined by the maximum value from one of two/three blind punched ngTMAs^®^ (which is equivalent to a blindly selected region of interest (ROI) rather than a hot spot selected in the whole slide). Only 29 patients out of 209 (13.9%) with NOS adenocarcinoma that had an ITB score <10 died of the disease, whereas 11 of 36 (30.5%) patients died of the disease when they had tumors with an ITB score ≥10 (*p* = 0.006). There was no significant correlation between ITB and overall survival (OS) (*p* = 0.441).

**Figure 5:**
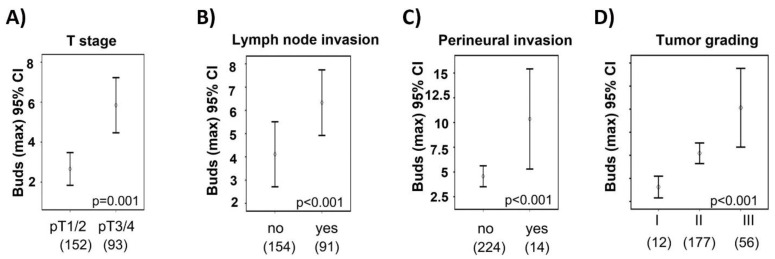
ITB score and clinicopathological parameters. Difference between the groups is shown, depending on the ITB score and (**A**) T category (T stage/UICC), (**B**) lymph node invasion/positive lymph nodes, (**C**) perineural invasion (not defined in 7 cases), and (**D**) tumor grading. The number of cases is shown in brackets. (**A**–**C**) Mann–Whitney U test; (**D**) Kruskal–Wallis test.

A high budding score was significantly associated with a higher tumor grade, higher pT stages, a higher lymph node metastasis, and a higher perineural invasion (Table 1, Figure 5A–D), but was not associated with a lymphatic invasion (Figure S1). Interestingly, the ITB score was not associated with the MSI vs. MSS status as a molecular marker, or with sex, age, venous invasion, or tumor localization (left- or right-sided colon).

For ITB, the median was calculated (cut-off >1), but this boundary seemed unsuitable for routine diagnostics (for further details, see Section S4). A univariate Cox regression showed that an ITB score of ≥10, the pT category, lymphatic invasion, venous invasion, and lymph node metastases had significant prognostic value (Table 2). When including all parameters that were significant in the univariate Cox analysis, only lymphatic invasion was confirmed as an independent prognostic factor. There was still a 1.9-fold risk for metastasis when the ITB score was high, but the significance was only marginal (Table 2).

### 2.6. ITB Evaluation in Histological Subtypes and the Correlation with Prognoses

**Figure 6:**
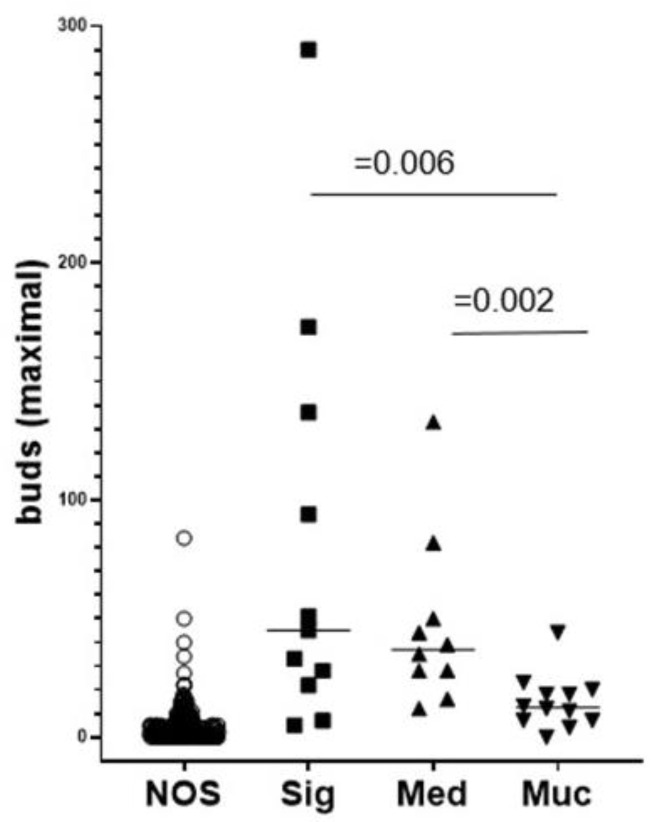
Comparison of NOS and histological subtypes regarding the distribution of max bud counts in each type of colon cancer using two-tailed, unpaired, and non-parametric Wilcoxon–Mann–Whitney testing. Calculated with SPSS Version 26, IBM (Armonk, New York, NY, USA). NOS compared with the subtypes of signet ring cell, medullary, and mucinous adenocarcinoma (*p* < 0.001). It can be seen that for NOS and mucinous carcinomas, most patients have low ITB scores. For signet ring cell and medullary carcinoma, however, most patients are in the range corresponding to high ITB scores.

We next evaluated ITB according to the three histological subtypes: signet ring cell, medullary, and mucinous adenocarcinoma. Interestingly, there were higher ITB scores with an extraordinarily high range for maximum scores in the subtype cohorts with signet ring cell and medullary carcinoma (Figure 6).

Each histological subtype seems to follow its own growth characteristics (e.g., in the comparisons of high and low scores for budding in the subtypes; see Figure S2).

We recognized the potential risk of predicting and extracting trends in the clinical data of those patients, according to which ITB also seemed to be associated with positive lymph nodes. Notably, higher sample numbers were necessary to perform statistically robust survival analyses.

### 2.7. Intratumoral Budding in Histological Subtypes and Clinical Outcomes

In the ITB evaluation, both the median and the maximum values from three evaluated ROIs were determined for each patient, whereby the best discriminatory power for each histological subtype was reflected by an unfavorable high ITB score.

In signet ring cell carcinoma, a high ITB score appeared as a cofactor with positive lymph nodes. By contrast, in medullary carcinoma, a high ITB score was associated with an unfavorable prognosis, regardless of lymph node status. A median of ≥20 was shown to be the optimal cut-off that resulted in the best delta for survival. Nevertheless, we deliberately chose a maximum bud score of ≥20 as the cut-off, which was, similarly, able to separate individuals in terms of survival. A maximum bud score is most conducive to its integration into clinical routines. However, more individuals are needed to define a reliable cut-off. This conspicuous finding seems to reflect the different growth characteristics and invasive behaviors of those histological subtypes compared to NOS tumors. Accordingly, ITB cut-offs determined for NOS do not appear to be appropriate for these subtypes. The different cut-offs and evaluation criteria between those subtypes and NOS explicitly result from the consideration of the underlying biology, different morphology, and growth patterns [26]. ITB scores, including their specific cut-offs, seem to be applicable in routine diagnostics since histological subtypes have to be reported according to the WHO classification of tumors [27]. For further information, see Figure S3.

### 2.8. The Comparison of the Mean Survival Rates between Histological Subtypes

In Table 3, the mean survival rates of patients with their corresponding histological subtypes and ITB scores were analyzed and sorted by descending survival. The best cut-off approach was chosen for the analysis. The patient cohorts were therefore divided into high and low budding for NOS tumors according to a maximum bud score of ≥10. The best cut-off for histological subtypes was a maximum bud score of ≥20. Within the subtypes, a lower ITB score indicated better survival (Table 3).

## 3. Discussion

Our study suggests a supporting role for ITB not only in the prognosis of patients with adenocarcinoma (NOS) but also in those with the analyzed histological subtypes based on uniform evaluation criteria.

There is a consensus on the importance of tumor buds, and studies on their biology and microarchitecture have been conducted.

Studies have shown PTB to be a relevant factor in the prognosis of patients with CRC by correlating it with lymphatic and venous invasion as well as lymph node metastases [4,12]. Similarly, the impact of ITB on patient prognosis has been determined [21,28]. However, tumor budding is still not regularly reported in clinical routines due to the lack of an internationally accepted uniform definition and studies suggest that tumor buds are not completely detached from tumor masses [20,29,30]. Mitrovic et al. demonstrated the need for established rules for tumor budding in their work [20]. PTB has been studied much more frequently in the literature, with significant differences in staining, evaluation, and counting criteria compared with ITB [20].

Unlike PTB, only a few studies have analyzed ITB using different approaches, but no histological subtypes have been analyzed. Lino-Silva et al., for example, analyzed ITB with different cut-offs (≤4 and ≥5 for low and high budding, respectively; HE) [31]. Marx et al. analyzed ITB using the TMA approach (IHC and HE) and the same bud cell count as we have used in our study here [28]. The ITBBC definition was used in both studies, which has actually been established for PTB. Lugli et al. defined different cut-offs (≤6 and >6 for low and high budding, respectively; IHC) in their studies on ITB [21].

In our study, we performed approximately 14,500 handmade annotations for ITB in NOS adenocarcinoma as well as in the subtypes of mucinous, signet ring cell, and medullary adenocarcinoma. We were primarily guided by morphology, which is reflected in the evaluation criteria we defined. Some hitherto published aspects of tumor budding (e.g., ≤4 cells) were included in the evaluation, as long as they agreed with the morphology. Based on annotations, we were able to designate cut-offs for NOS and histological subtypes, each based on a maximum of buds/core (ngTMA^®^) and the correlation with distant metastasis-free survival in NOS adenocarcinoma (see Figure 3 and Section 2.4), as well as a detailed definition of buds and a counting manual for ITB detection (see Section 4.4). We have hereby described the most detailed set of criteria, which distinguishes this study from previous studies.

The evaluation of ITB based on IHC is hardly comparable to PTB assessment using HE, despite sharing a similar cut-off for high budding. In the investigation of ITB, we believe that IHC will facilitate and improve tumor bud evaluation.

In some studies, by experts in the field, such as Bokhorst et al. and Haddad et al., the definition of buds is still under debate. In 2020, Bokhorst et al. published a study in which seven observers, as experts in the field of tumor budding, evaluated different bud candidates (PTB; AE1/AE3) [29]. There was complete agreement between all observers in 34% of bud candidates, which were classified as buds, poorly differentiated clusters (PDCs), or neither. Tumor buds were evaluated according to the ITBCC [4,29].

In 2021, Haddad et al. published a paper on a Delphi consensus study [31]. An international group of experts reached a consensus on whether the current ITBCC criteria should be expanded or modified. Pseudobuds, which are formed by inflammation and other influences and can be mistaken for tumor buds, were discussed [32]. The authors identified poorer visualized nuclei as a deficiency of the IHC and the significant role of the ITBCC criteria in tumor bud evaluation [32].

Results from Bokhorst et al. and Haddad et al. did not influence our 2021 evaluation. We agree with some decisions made by Bokhorst et al. in defining buds. On the other hand, some examples of the evaluated buds did not seem to meet our developed budding criteria.

Disagreement, in our view, exists with regard to the reported poorer visualization of nuclei by immunohistochemical staining in both studies due to nuclear truncation. This needs to be discussed, as the definition of buds based on HE, which has been suggested as a solution to address this issue, is also known to be adversely affected by underlying cutting artifacts. We showed possibilities for identifying ITB even when an obvious nucleus is lacking. These can be seen in the flowchart in Section 4.4.

In our study, ITB demonstrated itself as an independent and robust prognostic biomarker based on distant metastasis-free survival with *p* = 0.006 for NOS adenocarcinoma. In histological subtypes, the occurrence of ITB also seems to influence a patient´s prognosis.

The tumor center seems to be particularly appealing for tumor bud validation regarding the prognosis because in its center region the tumor may develop buds on its own (ITB) with less interference, e.g., by the immune system. As a speculation, we see intratumoral budding as an expression of an unfavorable characteristic, as the tumor shows disseminated growth (ITB) because it can and not because it has to (e.g., a response to immune cells or other “tumor-host interactions”) [33]. Indeed, an inverse correlation between the number of tumor buds and the presence of an immune response has been reported [33]. Interfering factors at the tumor center, such as the immune system, are mostly reduced and seem to not interfere with budding as much as they do at the invasion front [25]. This could also be due to a vast surface area of a growing tumor for interaction with, e.g., immune cells, and different rules might apply to this site. Those aspects are still under discussion and also need to be tested in the future.

Few studies to date have attempted to establish additional evaluation criteria for tumor budding when attempting to consider histological subtypes. The following exclusion criteria are mentioned for regarding cells as buds in the study of Mitrovic et al.: “glandular fragmentation associated with a marked acute inflammatory infiltrate”, “tumor fragments floating in mucin pools”, and “fragmented glands with surrounding retraction artifact” [20]. These mentioned aspects were also considered in our work (see Section 4.9).

In 2018, Takamatsu et al. performed an evaluation of PTB by IHC (AE1/AE3; bud definition ≤4 cells) and highlighted the utility of semi-automated computer-assisted methods. As in our study here, they used criteria for extra-glandular and extra-mucin locations [34]. Aside from this, no further morphological criteria were defined, and no histological subtypes were analyzed.

In 2020, the International Collaboration on Cancer Reporting (ICCR) released a guideline for the standardized reporting of inter alia colon carcinoma [35]. In this guideline, the criteria for diagnosing tumor budding are similar to those previously used in the literature. It is recommended that tumor budding should only be analyzed in non-mucinous and non-signet ring adenocarcinoma [35].

However, in the same year, the association between prognoses and PTB were presented, particularly in mucinous carcinoma, but without detailed evaluation criteria [36].

In 2021, tumor budding was finally considered in histological subtypes of colorectal carcinoma [5]. Using the ITBCC criteria, PTB in histological subtypes also appeared to affect survival, analogous to our thesis on ITB, but no specific morphological criteria were established [5].

This demonstrates the evolution of these criteria, whereby tumor budding (PTB) is taken into account in research and clinical routines. We believe that a similar development can be expected for ITB in the future. Cut-offs and established specific evaluation criteria including major histological subtypes of adenocarcinoma may support this development.

In regard to limitations, we did not apply a rigid scoring system, and only data of a single center cohort were analyzed. There was no significance in overall and disease-free survival, possibly due to the small size of the cohort. However, the association between the ITB score and metastasis-free survival was significant, which can be considered an important factor for patients. Despite our detailed description of bud detection, the experience of the pathologist remains necessary in the interpretation and evaluation of bud candidates. The cohort of patients with less frequently occurring adenocarcinoma subtypes was probably too small to allow for a robust significance level. Nevertheless, a trend in terms of survival became apparent. To achieve greater significance, ITB must be analyzed with a larger cohort (e.g., using a multi-center approach).

ITB has the potential to play a significant role in the prognosis of patients. In addition, the ITB analysis is both easy to implement and uniformly applicable. Therefore, ITB should be analyzed according to well-defined rules to strengthen its role and allow it to prevail in routine diagnostics based on stronger fundamental data.

As a long-term goal, we plan to develop a routine IHC-based AI following the ITB criteria (see Section 4.4 and flowchart). To this end, an algorithm trained on preliminary data has been published [37].

## 4. Materials and Methods

### 4.1. Cohort of Patients with Adenocarcinoma

Peritumoral and intratumoral budding was analyzed in 653 patients with primary colon cancer treated at the University Hospital of Erlangen between 2002 and 2010. The patients in the investigated cohort had typical pathological factors. Punches could only be evaluated in 245 out of the 653 available patients due to core loss or a lack of fulfillment of study criteria. Inclusion criteria required that all selected patients had at least two evaluable cores, were R0-resected, had not received neo-adjuvant therapy, and did not present distant metastases at the time of resection. Patient data were prospectively recorded from the hospital and included the diagnosis, age, and tumor grade as well as follow-up data, such as postoperative therapies, disease-free survival, recurrence-free survival, and overall survival.

### 4.2. Next-Generation Tissue Microarray Construction (ngTMA^®^)

The construction and analysis of TMAs were realized following the guidelines of next-generation tissue microarray construction (see Figure S4) [38]. All tissues were taken from the archive of the Institute of Pathology (University Hospital of Erlangen). All slides were scanned using a slide scanner (3DHISTECH PANNORAMIC 250 Flash II, 3DHISTECH Ltd., Budapest, Hungary). All TMAs were digitally analyzed using CaseViewer (Ver. 2.4., 3DHISTECH Ltd., Budapest, Hungary), and tumor buds were marked and numbered using the annotation tool of the program. The punch diameter was 0.6 mm (representing an area of 0.283 mm^2^). With at least two or three evaluable cores which met study criteria, the total area was similarly representative of the 0.785 mm^2^ at 20× (field of view: 25) proposed by the ITBCC [4]. These punches were performed with the intention of analyzing all samples using a double-blinded approach while avoiding checking whole slides for hot spots or ROIs.

### 4.3. Immunohistochemistry (IHC)

**Figure 7:**
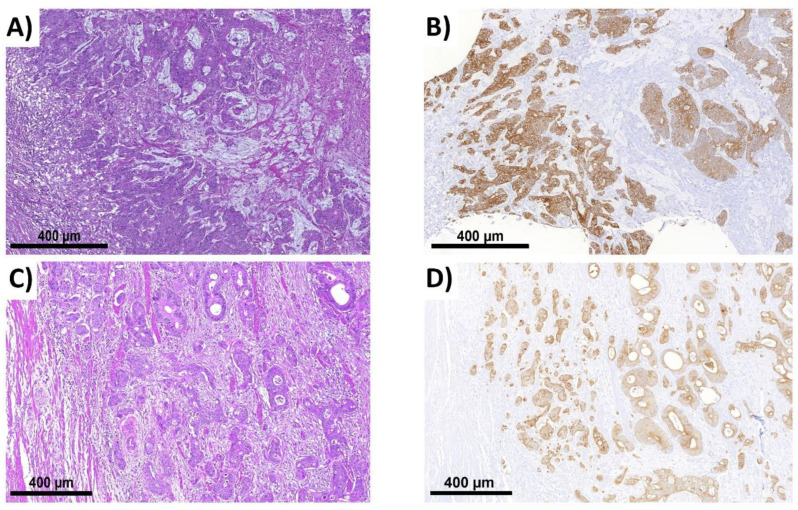
Comparison between HE and pancytokeratin (**A**–**D**). For technical data, see legend of Figure 1. All pictures are taken with 9× magnification. (**A**,**C**) A part of a section in HE; (**B**,**D**) corresponding areas to (**A**,**C**) in pancytokeratin. Even at this magnification, a few bud candidates can be estimated by visual inspection based on IHC.

For improved differentiation between inflammatory cells and those decomposing the tumor, the usage of pancytokeratin staining is recommended because tumor buds can be more easily found compared with using HE [22]. Punches of NOS adenocarcinoma and histological subtypes were stained using the antibodies AE1/AE3 according to the manufacturer’s recommendations (Zytomed Systems GmbH, Berlin, Germany), (Figure 7) [39]. The antibody for AE1 recognizes the acidic (type I) cytokeratins 10, 15, 16, and 19. The antibody of the AE3 clone recognizes all known basic (type II) cytokeratins, i.e., CK1 to 8. Antibodies were used at a dilution of 1:40. Sections were first pretreated with a citrate buffer at pH 6.0 and incubated at a working dilution of 1:100 for 30 min according to the manufacturer’s recommendations.

### 4.4. ITB Analysis Criteria

#### 4.4.1. General Information

The tumor bud analysis was partly based on the ITBCC 2016 criteria [4]. Based on the literature up to 2019, there are different staining and evaluation criteria. We have attempted to choose, implement, and expand upon the criteria that were the best in terms of successful identification. The vast majority of studies set the maximum cell count of a bud to ≤4. We adopted this in our study. All 14,530 tumor buds were annotated by hand; 3709 of them were of histological subtypes, 2327 were from tumor centers of whole slide images (WSI), and 2585 were from the invasion front. Furthermore, 3270 were from the TMAs of the tumor center, and 2639 were from the TMAs of the invasion front. All annotations were cross-checked by two experts (P.P.F. and C.G.), and a high quality of evaluation, interobserver reliability, and a concordance of 96% were achieved. Virtual meetings were regularly held where tumor buds were discussed.

**Figure 8:**
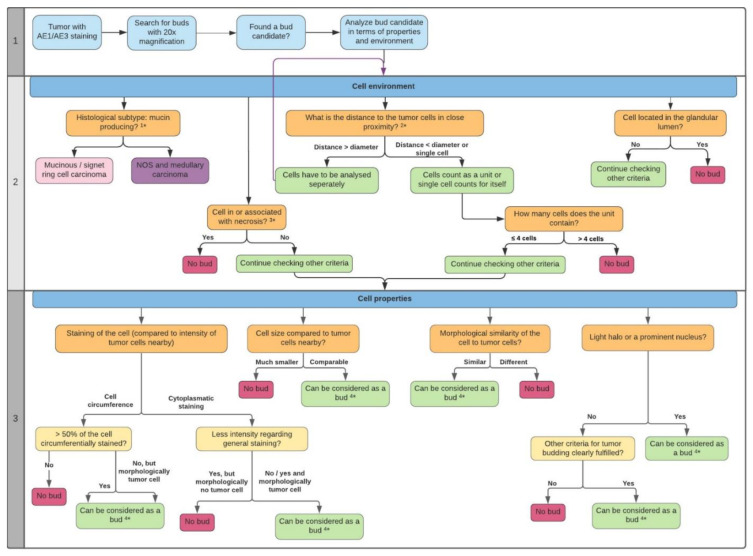
Flowchart to detect tumor buds. In the scheme, all relevant criteria are shown in Section 1, Section 2 and Section 3 regarding aspects for single cells and bud candidates and for their surroundings nearby. The flowchart was constructed using Lucidchart (2021 Lucid Software Inc., Amsterdam, Netherlands) and using a template for UML activity diagrams. Additional comments are provided in Section 4.4.2, Section 4.4.3 and Section 4.4.4. The footnotes of this scheme are resolved in Section 4.4.3 and Section 4.4.4.

Throughout this the process, refinements in criteria were achieved, a flowchart was created, and tumor buds were retrospectively redefined. During our evaluation, we focused on the criteria of the bud or cell environments as well as the cell properties. This led to a manual for detecting and defining tumor buds (Figure 8).

#### 4.4.2. Preparatory Steps

As shown in the first section of Figure 8, tissue should be stained with pancytokeratin (AE1/AE3) and analyzed at 20×. Once a bud candidate is found, properties related to the cell itself and its environment have to be analyzed.

#### 4.4.3. Cell Environment

As shown in the second section of Figure 8 (the cell environment), aspects of the environment have to be included in the analysis. The histological subtype should be identified. The flowchart should be followed for NOS adenocarcinoma.

^1*^ Further evaluation criteria for medullary, mucinous, and signet ring cell carcinoma are explained in the corresponding passage (Section 4.9).

^2*^ Distance criteria are defined according to the average diameter of the surrounding tumor cells. If the distance between bud candidates and tumor cells is larger than the average diameter, they must be individually analyzed with regard to all budding criteria. If the distance is smaller than the average diameter, the cells must be grouped together and counted as a unit.

To analyze a cell unit, the number of cells therein must be verified. If it contains ≤4 cells, this group can be considered a single bud as long as the other criteria are fulfilled. A group of ≥5 cells exceeds the maximum cell number as per the definition. Candidates located outside a lumen fulfill another bud criterion and should be further analyzed.

^3*^ Attention should be paid to necrosis and its proximity. If the bud candidate is part of, or close to, necrosis, it cannot be considered a bud. This rule also includes cases of comedonecrosis, as seen in medullary carcinoma. If a ruptured gland containing comedonecrosis is present, tumor cells scattered in the proximity can only be considered as buds if they fulfill the criteria for being located at a sufficient distance.

#### 4.4.4. Cell Properties

If all criteria for the cell environment are fulfilled, it is necessary to check all properties, as described in the third section of Figure 8 (cell properties). This includes continuous, circumferential staining, although this may be an artifact due to the asymmetric distribution of cytoplasm (see Section 2.2). With a circumferential staining of more than 50% or a morphologically clear presence of a tumor cell (also with a circumferential staining of <50%) the candidate can be considered a bud. Weakly stained cells should also be considered buds unless they are morphologically not tumor cells. Vice versa, more or less positive cells for AE1/AE3 that seem to not be tumor cells, like macrophages that have phagocytized epithelial/tumor cell fragments, or other related inflammatory cells, for instance, should not be considered any further for ITB. This should also be taken into account in the case of morphologically avital or apoptotic tumor cells. Furthermore, the cell size of the candidate should be compared with the average size of the remaining tumor cells nearby. If the cell is significantly smaller than the overall average, it should not be considered a bud. In addition, there must be a morphological similarity between the bud candidate and the rest of the tumor. If there is a large morphological difference, the candidate should not be considered a bud. It is important to assess whether the cell has a visible nucleus. If not, then whether it can be considered a bud or not depends on whether the other budding criteria are met. Nuclear truncation must be considered (Figure 7). If the candidate appears to be a bud in all other aspects, it should be considered a bud, even if it does not have a well-defined nucleus due to cell truncation effects.

^4*^ All criteria have to be fulfilled for the cell or group of cells to be considered a bud.

### 4.5. TMA Analysis

Three punches of the tumor center and an invasive margin were each separately analyzed for every single patient. Only individuals with 2/3 or 3/3 sufficient punches were used for the evaluation. TMA punches were excluded from further analyses if there was no vital tumor, the tissue was not stained sufficiently, or the tissue was lost during the staining process. The number of buds and clinical data were documented. We initially hypothesized that tumor budding at the invasive margin is significantly correlated with distant metastasis-free survival, along with the ngTMA^®^ punches. However, results at the invasive margin showed no sufficient significance for patient prognosis. Therefore, we focused on ITB instead.

### 4.6. The ITB Analysis

Three blinded and randomly selected punches from the tissue block within the tumor areas were analyzed. The cores were taken unselected, without any indication regarding the location or hot spot of tumor buds. They were analyzed with the same criteria as those for the invasive margin. A major problem was posed by dedifferentiated tumors, which had poor pancytokeratin staining due to the loss of certain cell characteristics. Those cases were carefully analyzed.

According to routine diagnostics, when a preliminary assessment by HE provides an impression of the tumor, considers its differentiation, and shows special aspects such as the necrotic formations or ruptured glands, HE can be used. The best section with representative tumor tissue can be selected for IHC. ITB is evaluated afterwards by microscope, based on the described criteria (Figure 8). Scientists were blinded in this study to avoid the selection of hot spots and were also blinded in the budding analysis regarding clinical data.

### 4.7. The WSI Analysis

In the course of our evaluation, we analyzed the WSI from selected patients. We wanted to analyze as many buds as possible to strengthen our criteria and refine the flowchart for ITB. Thus, we selected three spots with a 1 mm diameter in each tumor center for a maximum number of buds in one area for evaluation.

### 4.8. Patients with Histological Subtypes Other Than Adenocarcinoma NOS

**Figure 9:**
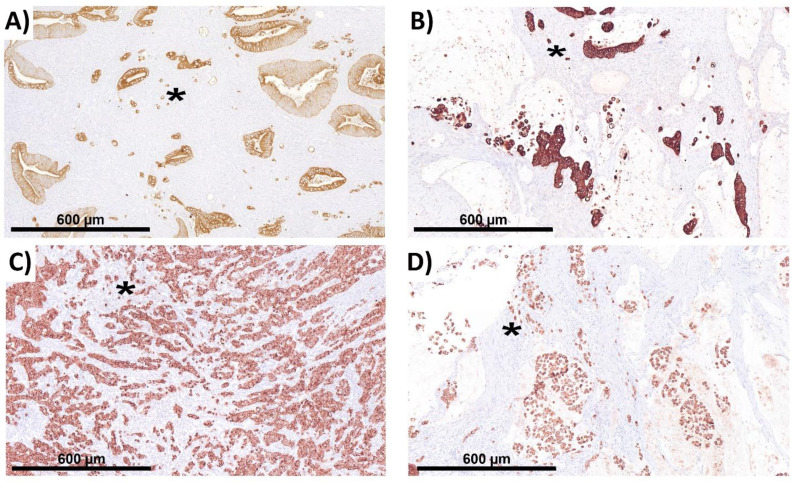
Histological subtypes. For technical data, see legend in Figure 1. All images were taken at 10× magnification. All 4 analyzed subtypes are shown in A–D. (**A**) NOS, (**B**) mucinous carcinoma, (**C**) medullary carcinoma, and (**D**) signet ring cell carcinoma. Exemplary buds are marked with an asterisk (*), that are located in the stroma and that fulfil the given criteria for ITB.

In the first evaluation of ITB in NOS, it was suspected that budding behavior, considering the distinct biology, is different in certain histological subtypes. Therefore, in a second approach, we analyzed the histological subtypes separately. This cohort included 12 patients with mucinous, 11 with signet ring cell, and 10 with medullary carcinoma. These patients were treated between 2000 and 2010 at the University Hospital of Erlangen. Histological subtypes were selected (the R0 status without neo-adjuvant therapy and no distant metastases), and the ITB analysis was conducted with blinding for the clinical data (for an overview of histological subtypes, see Figure 9).

### 4.9. The ITB Analysis in Histological Subtypes

For the evaluation of the histological subtypes, we purposely did not use ngTMA^®^. The WSI with AE1/AE3 were used to evaluate as many representative areas as possible. Three spots with a diameter of 1 mm from the tumor center were selected. Criteria included the following: buds should not be located directly at the mucosa or at the invasion front, and spots had to contain vital tumor tissue and be clear of artifacts (previous biopsies, heat artifacts, etc.). Therefore, these were not necessarily areas with the highest tumor cell or bud densities. Each spot contained buds and connective tissue and was located in the tumor center outside necrosis or any mucus. However, by encountering the specifics, the criteria for subtypes were extended and adapted.

#### 4.9.1. Medullary Carcinoma

In the case of medullary carcinoma, attention has to be paid to the distance of tumor cells, especially between weakly stained tumor cells. Dedifferentiated adenocarcinoma have altered antigen expression with, e.g., less CDX2 and/or less cytokeratin [38]. To reconstruct the relevance of weakly stained cells, we repeated the staining with the set of medullary carcinoma with a 1/2 dilution. We placed them in the same spots and counted for buds. All bud candidates would have been counted here as before. This supports the hypothesis that the weakly stained tumor cells are as important as strongly stained tumor cells in IHC.

#### 4.9.2. Mucinous and Signet Ring Cell Carcinoma

For these subtypes, it is important to note that, due to their produced extracellular mucin, the cells in the mucus lakes cannot be considered tumor buds, as previously described [20]. Only cells clearly located in the connective tissue represent a bud. In addition, the remaining criteria of the flowchart must be fulfilled. If a cell lies at the edge of a mucus lake, it is necessary to see where the main focus of the cell or cell group (only ≤4 cells) is located. If this main focus is located in the stroma, then it can be evaluated to be a bud as long as all other criteria are fulfilled. If the main focus is located in the mucus lake, it cannot be considered a bud.

### 4.10. The Statistical Analysis

The statistical analysis was performed using SPSS Version 26, IBM (Armonk, New York, NY, USA). A univariate Cox regression was conducted to determine the risk of distant metastases for intratumoral budding and other clinicopathological parameters, and *p* < 0.05 was considered to indicate statistically significant differences. All variables where *p* < 0.05 in the univariate analysis were combined into a multivariate analysis to determine the independent prognostic factors. A best cut-off approach was used both for NOS adenocarcinoma and for histological subtypes. The dependence of clinical data with a median bud count of two/three cores was also analyzed. High and low ITB was correlated with distant metastasis-free survival and with overall survival. The data distribution of the results was assessed using the Kolmogorov–Smirnov test. The histogram did not show a normal distribution of data, so a Mann–Whitney U test was used.

## 5. Conclusions

In summary, ITB appears to be a routinely useful prognostic marker in the diagnostics of colon cancer. Our study provides the much-needed uniform rules for ITB evaluation as part of our work, which are consistent and applicable. We not only looked at tumor buds as just cells but also related them to the rest of the tumor and their environment. We sought to take a deeper look at ITB and are the first to develop a manual for detecting and defining tumor buds.

We also performed analyses of ITB for the most common histological subtypes in the literature and described the specific analysis criteria. Following this, detailed ITB guidelines of daily routines were analyzed. The scoring system was demonstrated in independent and blinded punches, which allowed for sufficient evaluation via the ngTMA^®^ approach and in routine settings. We also demonstrated the suitability of AE1/AE3 as a biomarker for ITB and reliably combined it with the criteria catalogue (Figure 8). Furthermore, in ongoing experiments, ITB characteristics and annotations are subjects for AI training which may be used as a future tool for tumor bud definition in diagnostics. We believe that, based on this work, tumor budding is taking a new direction, and detailed ITB evaluations can be successfully used by pathologists. This study contributes a new standard for defining ITB and its distribution, not only in NOS tumors but also in the analyzed histological subtypes. Regarding the ITB evaluation in colon carcinoma subtypes, a foundation is laid for further studies (e.g., a multicenter study).

## Figures and Tables

**Table 1:** Patient characteristics and associations with tumor budding score, *n* = 245.

Feature		Frequency *n* (%)	ITB < 10	ITB ≥ 10	*p*-Value
Sex	Male Female	152 93	126 (82.9) 83 (89.2)	26 (17.1) 10 (10.8)	0.173
Age	≤65 years >65 years	94 151	79 (84) 130 (86.1)	15 (16) 21 (13.9)	0.659
Localization	Left-sided Right-sided	108 137	94 (87.0) 115 (83.9)	14 (13.0) 22 (16.1)	0.497
Tumor grading	G1/2 G3/4	189 56	169 (89.4) 40 (71.4)	20 (10.6) 16 (28.6)	0.001
Stage UICC	I/II III/IV	152 93	141 (92.8) 68 (73)	11 (7.2) 25 (27)	<0.001
pT category	pT1/2 pT3/4	70 175	66 (94) 143 (81.7)	4 (6) 32 (18.3)	0.012
Primary metastasis *	pM0 pM1	231 14	198 (85.7) 11 (79)	33 (14.3) 3 (21)	0.464
pN category	pN0 pN1,2	154 91	143 (92.9) 66 (73)	11 (7.1) 25 (28)	<0.001
Lymphatic invasion	No	176	157 (89.2)	19 (10.8)	
	Yes	69	52 (75)	17 (25)	0.006
Venous invasion	No	235	201 (85.5)	34 (14.5)	
	Yes	10	8 (80)	2 (20)	0.628
Perineural invasion	No	224	196 (87.5)	28 (12.5)	
	Yes	14	8 (57.1)	6 (42.9)	0.010
MSS/MSI status	MSS	198	169 (85.4)	29 (14.6)	
	MSI	47	40 (85)	7 (15)	0.966

* Primary metastasis: pM1 indicates individuals with complete resection (local and distant R0).

**Table 2:** Univariate and multivariate Cox regression analyses for risk of metastasis (*n* = 245).

Parameter	Variable	Hazard Ratio	95% CI	*p*-Value
*Univariate analysis*				
Age (years)	>65	0.932	0.498–1.746	0.826
pT category	pT3/4	4.167	1.483–11.708	0.007
Tumor grade	G3/4	1.956	1.021–3.747	0.043
Lymphatic invasion	L1	4.824	2.558–9.098	<0.001
Venous invasion	V1	3.344	1.188–9.409	0.022
Tumor localization	Left-sided	1.476	0.771–2.828	0.240
Lymph node metastases	pN1,2	2.686	1.434–5.032	0.002
ITB score	≥10	2.542	1.269–5.090	0.008
*Multivariate analysis*				
pT category	pT3,4	2.354	0.802–6.906	0.169
Lymphatic invasion	L1	3.519	1.701–7.282	0.001
Venous invasion	V1	2.095	0.731–6.009	0.169
Lymph node metastases	pN1,2	1.099	0.524–2.307	0.802
ITB score	≥10	1.972	0.949–4.098	0.069

**Table 3:** ITB scores depending on histological subtypes and a comparison regarding metastasis-free survival (ranking).

Subtype	ITB Score *	Number of Patients (*n*) ^1^	Survival (Months) ^2^	Delta (Months) ^3^	Ranking ^4^
NOS	Low	210	114.0	11.0	2
NOS	High	35	103.0		3
Medullary Ca	Low	2	116.3	48.6	1
Medullary Ca	High	8	67.7		5
Signet ring cell Ca	Low	2	74.1	47.6	4
Signet ring cell Ca	High	9	26.4		8
Mucinous Ca	Low	9	65.3	33.7	6
Mucinous Ca	High	3	31.6		7

* Best cut-off for signet ring cell, mucinous, and medullary carcinoma: ITB max score ≥20; for NOS: ITB max score ≥ 10. ^1^ The total number of patients: for NOS, 245; for medullary carcinoma, 10; for signet ring cell carcinoma 11; for mucinous carcinoma, 12. ^2^ Metastasis-free survival in months (based on full days, and primary R0 status local and distant). ^3^ Delta is the difference in survival between the group with low ITB and the one with high ITB. ^4^ Ranking comparing histological subtypes in terms of favorable prognosis in terms of survival in months. Patients with medullary carcinoma and low ITB seem to show the best survival. Patients with signet ring cell carcinoma and high ITB, on the other hand, seem to have the worst prognosis. For more detailed information about patient information and median cut-offs, see Figure S3.

## Data Availability

Represented data are publicly archived datasets. For further information, please contact the corresponding author.

## Supplementary Materials

The following are available online at https://www.mdpi.com/article/10.3390/ijms222313108/s1. References [19,40,41,42,43,44] are cited in the supplementary materials.

## Abbreviations

AE1/AE3	cytokeratin cocktail for detecting different cytokeratins (CK1-8,10,14-16 and 19)
AI	artificial intelligence
CDX2	caudal-related homeobox gene 2
CK	cytokeratin
COST	Cooperation in Science and Technology
CRC	colorectal cancer
EMT	epithelial-to-mesenchymal transition
FAU	Friedrich-Alexander-University
FFPE	formalin-fixed paraffin-embedded
FISH	fluorescence in situ hybridization
HE	hematoxylin-eosin
ICCR	International Collaboration on Cancer Reporting
IHC	immunohistochemistry
ITB	intratumoral budding
ITBCC	International Tumor Budding Consensus Conference
MSS/MSI	microsatellite stability/instability
ngTMA	next-generation tissue microarray
NOS	no other specified
OS	overall-survival
PDC	poorly differentiated clusters
pH	pondus hydrogenii
PTB	peritumoral budding
ROI	region of interest
UICC	Union internationale contre le cancer
WHO	World Health Organization
WSI	whole slide image
