# Supplementary material for: Novel Criteria for Intratumoral Budding with Prognostic Relevance for Colon Cancer and Its Histological Subtypes
Source: Int J Mol Sci. 2021 Dec 3;22(23):13108. doi: 10.3390/ijms222313108 (PMC8658236; doi:10.3390/ijms222313108)
Supplement: Supplementary file 1 [file ijms-22-13108-s001.zip › ijms-1465704-supplementary.pdf]

Supplementary section 1 - E-cadherin and APAF1

Zlobec et al. tried to find out whether tumor budding is affected by the loss of specific proteins [40]. They found out that the "loss of expression of intercellular adhesion molecule E-cadherin and tumor suppressing APAF-1 were independent predictors of budding". It was shown that the loss of AFAP-1 correlates with stronger budding, peritumoral lymphocytes and shorter survival time. The loss of E-cadherin is also linked to a higher amount of buds and peritumoral lymphocytes. Poor survival with E-cadherin loss was only shown with the co-occurrence of APAF-1 loss and tumor-infiltration of lymphocytes [40]. No subtypes were analyzed.

Supplementary section 2 - EMT

In cancer there are different inducers of EMT such as hypoxia or cytokines. The loss of the previously mentioned E-cadherin in tumor buds, the increase of mesenchymal markers like vimentin and N-cadherin and the loss of polarized function of epithelial cells lead to a deficit of cell to cell-contact and the switch of epithelial cells into mesenchymal phenotypes [19]. They lose contact to the tumor and are able to start migration. EMT and the reverse process mesenchymal-epithelial-transmission (MET) found to be "not all-or-none responses". They are "multi-state processes, ranging from purely epithelial to purely mesenchymal via one or several intermediate phenotypes" [19]. Thus, intermediate phenotypes are referred to as partial EMT. There is not enough evidence for a connection between tumor budding and EMT but contrary to this there seems to be more evidence about the connection to partial EMT [19]. On the other hand, studies like Yamada et al. claim that "EMT-related proteins play a minor role in forming tumor buds" [41].

Supplementary section 3 – Immune response

In their paper Galon and Pagès from France showed that disease-free and overall-survival correlate with "number, type and location of tumor immune infiltrates in primary tumors" as prognostic factors [42]. With the observations an Immunoscore can be established "based on the numeration of two lymphocyte populations (CD3/CD45RO, CD3/CD8 or CD8/CD45RO), both in the [center] CT and in the [invasive margin] IM of tumors, as a clinically useful prognostic marker" [42,43]. It ranges from Immunoscore 0 containing low densities for CD8+ and CD3+ immune cells to Immunoscore 4 with high densities in both regions of TC and IM of those immune cells. Once the tumor gets detected, the adaptive host immune system seems to play an important role in preventing tumor recurrence. This would explain why patients with low Immunoscores (I 0 and I 1) experienced higher recurrence and poor survival compared to those with high Immunoscores. Combined with standard tumor staging they could have benefited from adjuvant therapy [43]. Also T cells with their characteristic long-lasting antitumor capacity could be fundamental for a long-term tumor immunity [42]. The impact of the immune system on cancer has not only been shown in colorectal cancer but also in other entities [44]. Unfortunately Galon and Pagès did not investigate the patients´ Immunoscores in terms of tumor budding.

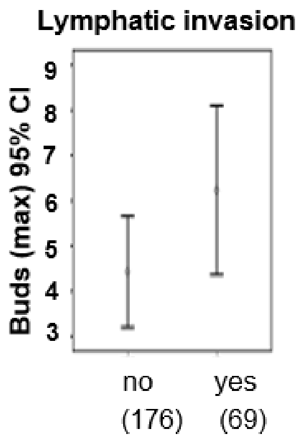

Figure S1. ITB score and lymphatic invasion. The difference between the groups is shown, depending on the ITB score and lymphatic invasion. In brackets number of cases are shown. Mann-Whitney test.

Supplementary section 4 – ITB and median score

For ITB, median of > 1 was statistically significant in univariate (p = 0.02) and multivariate (p = 0.042) analyses. However, we consider a differentiation between the tight boundary of a median of ≤ 1 and > 1 to be not suitable for routine diagnostics. The approach with the highest maximum therefore appears to be more suitable.

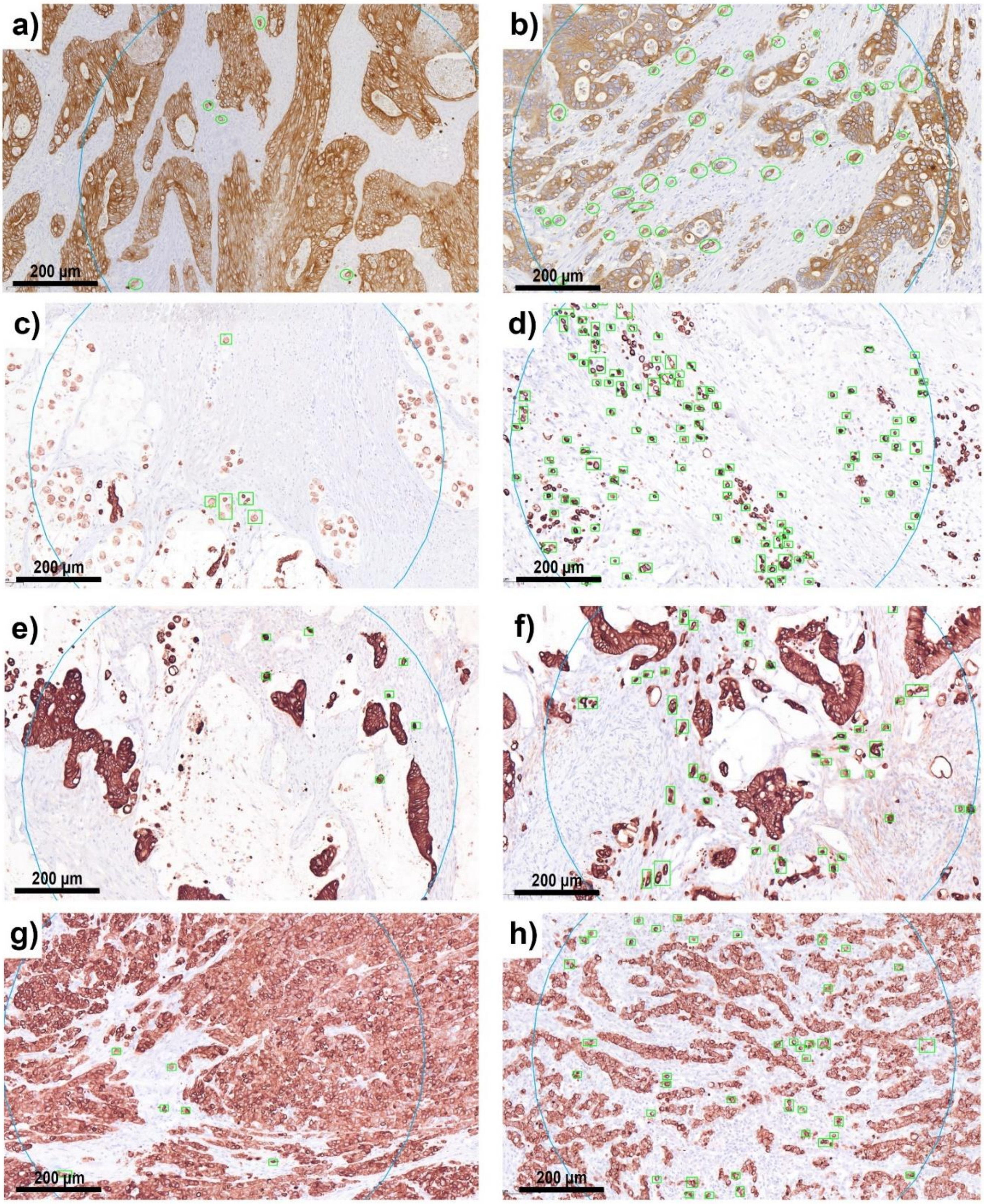

Figure S2. Comparison of high and low ITB areas **(a-h)**. For technical data, see legend Figure 1. Figure **a)-h)** taken with 15x magnification. Defined tumor buds are marked in green. Each row shows the comparison between a ROI with low and high ITB respectively for: **a, b)** NOS tumors, **c, d)** signet ring cell carcinoma, **e, f)** mucinous carcinoma and **g, h)** medullary carcinoma.

a)

Patient Subtype ID	Median	pT class.	Lymph node +	Survival (months)	Mean survival (months)
101	142	3	39	1,8	
102	256	4	20	28,5	
103	20	4	4	32,3	
104	91	4	12	3,3	27,5
105	41	3	2	91,4	
106	122	3	13	15,9	
107	30	3	0	1,8	
108	30	3	0	44,8	
109	4	4	2	49,0	
110	11	3	0	17,9	55,3
111	6	3	5	99,1	
					Total: 35,1

b)

Patient Subtype ID	Max	pT class.	Lymph node +	Survival (months)	Mean survival (months)
101	173	3	39	1,8	
102	290	4	20	28,5	
103	28	4	4	32,3	
104	94	4	12	3,3	
105	51	3	2	91,4	26,4
106	137	3	13	15,9	
107	33	3	0	1,8	
108	45	3	0	44,8	
110	22	3	0	17,9	
109	5	4	2	49,0	74,1
111	7	3	5	99,1	
					Total: 35,1

c)

Patient Subtype ID	Median	pT class.	Lymph node +	Survival (months)	Mean survival (months)
201	24	3	3	27,0	
202	38	3	0	88,7	
203	34	3	0	75,8	54,1
204	78	3	5	23,2	
205	108	3	0	48,7	
206	26	3	0	61,1	
207	15	3	0	99,5	
208	14	5	0	112,5	112,5
209	12	6	0	104,8	
210	10	2	1	133,1	
					Total: 77,4

d)

Patient Subtype ID	Max	pT class.	Lymph node +	Survival (months)	Mean survival (months)
201	39	3	3	27,0	
202	50	3	0	88,7	
203	44	3	0	75,8	
204	82	3	5	23,2	
205	133	3	0	48,7	67,7
206	28	3	0	61,1	
208	28	5	0	112,5	
209	35	6	0	104,8	
207	16	3	0	99,5	116,3
210	12	2	1	133,1	
					Total: 77,4

e)

Patient Subtype ID	Median	pT class.	Lymph node +	Survival (months)	Mean survival (months)
301	20	2	0	70,8	
302	34	4	6	13,4	31,6
303	22	3	10	10,6	
304	10	4	0	75,7	
305	15	4	7	36,0	
306	6	3	0	69,9	
307	4	3	0	79,7	
308	9	3	0	65,0	65,3
309	5	3	0	63,9	
310	0	3	0	88,1	
311	8	3	4	37,1	
312	8	2	2	72,1	
					Total: 56,9

f)

Patient Subtype ID	Max	pT class.	Lymph node +	Survival (months)	Mean survival (months)
301	20	2	0	70,8	
302	44	4	6	13,4	31,6
303	23	3	10	10,6	
304	18	4	0	75,7	
305	18	4	7	36,0	
306	7	3	0	69,9	
307	4	3	0	79,7	
308	13	3	0	65,0	65,3
309	7	3	0	63,9	
310	0	3	0	88,1	
311	11	3	4	37,1	
312	12	2	2	72,1	
					Total: 56,9

Figure S3. Tables of survival in histological subtypes **a-f**. The tables are divided according to median on the left (a, c, e) and maximum value on the right (b, d, f). Patients marked in red are above the respective cut-off. The patients marked in green are below the respective cut-off. There is one ID per subtype (Patient Subtype ID), which can be used to infer the patient. Subtype IDs: 1 signet ring cell, 2 medullary and 3 mucinous carcinoma. These are individuals that have been reassigned a subtype ID according to their subgroup. Thus they were anonymized. **a, b)** Data of signet ring cell carcinoma. **a)** Cut-off: median ≥ 20. 3 of 11 patients were below and 8 of 11 above cut-off. Mean survival of patients with low ITB was 55,3 months and of patients with high ITB was 27,5 months. This results in a delta of 27,8 months. **b)** Cut-off: maximum ≥ 20. 2 of 11 patients were below and 9 of 11 above cut-off. Mean survival of patients with low ITB was 74,1 months and of patients with high ITB was 26,4 months. This results in a delta of 47,6 months. **c, d)** Data of medullary carcinoma. **c)** Cut-off: median ≥ 20. 4 of 10 patients were below and 6 of 10 above cut-off. Mean survival of patients with low ITB was 112,5 months and of patients with high ITB was 54,1 months. This results in a delta of 58,4 months. **d)** Cut-off: maximum ≥ 20. 2 of 10 patients were below and 8 of 10 above cut-off. Mean survival of patients with low ITB was 116,3 months and of patients with high ITB was 67,7 months. This results in a delta of 48,6 months. **e, f)** Data of mucinous carcinoma. **e)** Cut-off: median ≥ 20. 9 of 12 patients were below and 3 of 12 above cut-off. Mean survival of patients with low ITB was 65,3 months and of patients with high ITB was 31,6 months. This results in a delta of 33,7 months. **f)** Cut-off: maximum ≥ 20. 9 of 12 patients were below and 3 of 12 above cut-off. Mean survival of patients with low ITB was 65,3 months and of patients with high ITB was 31,6 months. This results in a delta of 33,7 months.

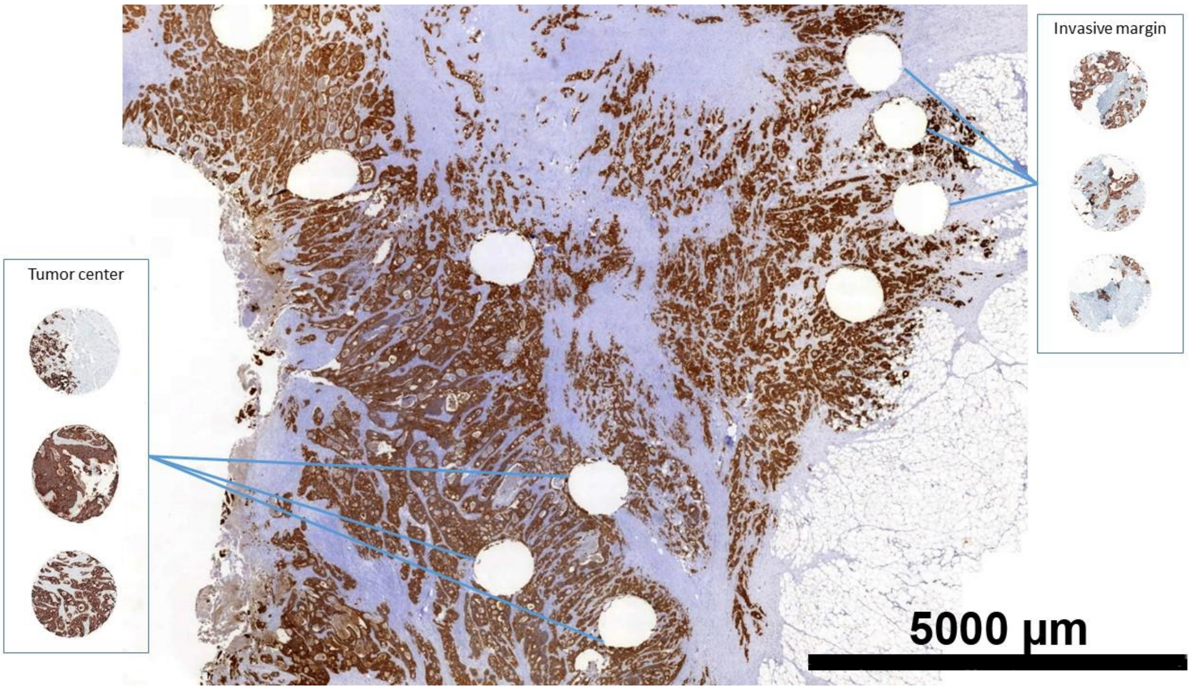

Figure S4. WSI with its TMA-punches. For technical data, see legend Figure 1. The image was taken at 1.1x magnification. The sampling locations for the ngTMA® punches can be seen in the WSI. These were randomly selected. TMA punches taken from the tumor center are shown on the left and those from the invasion front are shown on the right side (inserts).
